# Supplementary material for: One year prevalence of psychotic disorders among first treatment contact patients at the National Psychiatric Referral and Teaching Hospital in Uganda
Source: PLoS One. 2020 Jan 29;15(1):e0218843. doi: 10.1371/journal.pone.0218843 (PMC6988969; doi:10.1371/journal.pone.0218843)
Supplement: Attachment — Submitted filename: Review Comments.docx [file pone.0218843.s002.docx]

Thank you for the opportunity to review this paper.

It is a necessary piece of research and has been written fairly well. However, there are some concerns that need to be addressed before proceeding to the next level.

1. The authors have mentioned the bed capacity of the study site but have not described the bed occupancy. This would be helpful in putting the information provided into context
2. It is not clear how diagnosis in the patient charts was confirmed since there are many cadres (with varying expertise) involved in assigning a diagnosis.
3. It is not clear if the files reviewed were for outpatients or inpatients or both.
4. How many patients does the hospital see annually in outpatient and inpatient units/wards?
5. Most patients become religious as a way of coping with the unusual psychiatric experiences as opposed to change of religion. Was the religious affiliation confirmed by duration in the assigned religious group?
6. The study site/hospital is a referral hospital but rhe study refers to first contact. How many of the patients were accessing the hospital for the first time and how many of them had been referred from the lower health units?
7. In the results section, the prevalence is recorded as 62.7% but in discussion, it is 67%. Which is the correct prevalence for psychotic disorders?
8. Low numbers of patients below 18years should be linked with duration of psychotic symptoms before speculating about late presentation to hospital. Otherwise, it would be a speculation.
9. Which diagnostic criteria were used to diagnose psychotic disorders? This is not clear from the write up.
